# Likeability and Expert Persuasion: Dislikeability Reduces the Perceived Persuasiveness of Expert Evidence

**DOI:** 10.3389/fpsyg.2021.785677

**Published:** 2021-12-23

**Authors:** Mariam Younan, Kristy A. Martire

**Affiliations:** School of Psychology, The University of New South Wales, Kensington, NSW, Australia

**Keywords:** expert, juror decision-making, evidence evaluation, likeability, credibility, persuasion

## Abstract

With the use of expert evidence increasing in civil and criminal trials, there is concern jurors' decisions are affected by factors that are irrelevant to the quality of the expert opinion. Past research suggests that the likeability of an expert significantly affects juror attributions of credibility and merit. However, we know little about the effects of expert likeability when detailed information about expertise is provided. Two studies examined the effect of an expert's likeability on the persuasiveness judgments and sentencing decisions of 456 jury-eligible respondents. Participants viewed and/or read an expert's testimony (lower vs. higher quality) before rating expert persuasiveness (via credibility, value, and weight), and making a sentencing decision in a Capitol murder case (death penalty vs. life in prison). Lower quality evidence was significantly less persuasive than higher quality evidence. Less likeable experts were also significantly less persuasive than either neutral or more likeable experts. This “penalty” for less likeable experts was observed irrespective of evidence quality. However, only perceptions of the foundational validity of the expert's discipline, the expert's trustworthiness and the clarity and conservativeness of the expert opinion significantly predicted sentencing decisions. Thus, the present study demonstrates that while likeability does influence persuasiveness, it does not necessarily affect sentencing outcomes.

## Introduction

Expert evidence is ubiquitous in modern civil and criminal trials (Gross, 1991; Diamond, 2007; Jurs, 2016). Jurors involved in legal proceedings must assess the value of expert opinions to inform consequential decisions affecting lives and liberty. However, these assessments are sometimes mistaken, threatening the administration of justice, and contributing to unsafe trial outcomes (Innocence Project, 2021).

The Elaboration Likelihood Model (ELM) of persuasion is an information-processing model that has been used to understand jury decision-making about expert evidence (Petty and Cacioppo, 1986; McAuliff et al., 2003). This model suggests that jurors may struggle to accurately distinguish between low- and high-quality expert opinions because of the cognitive demands involved in the task (Petty and Cacioppo, 1986; Greene and Gordan, 2016). According to ELM, limited cognitive resources and insufficient knowledge increase reliance on readily accessible but potentially irrelevant, peripheral aspects of a message (Petty and Cacioppo, 1984, 1986; San José-Cabezudo et al., 2009; Salerno et al., 2017). This theory is supported by evidence suggesting that when information is unfamiliar, highly technical or complex—as is often the case for expert opinions—juror evaluations of credibility and persuasiveness may be swayed by superficial features of the expert and their evidence (Chaiken, 1980; Heuer and Penrod, 1994; Shuman et al., 1994; Cooper et al., 1996; Schuller et al., 2005; Ivković and Hans, 2006; Daftary-Kapur et al., 2010; Bornstein and Greene, 2011; Neal, 2014; Maeder et al., 2016). Expert likeability is one peripheral cue that may affect perceptions of persuasiveness.

“Likeability” refers to the extent to which an expert presents as friendly, respectful, well-mannered, and warm (McAdams and Powers, 1981; Kerns and Sun, 1994; Levin et al., 1994; Gladstone and Parker, 2002; Neal and Brodsky, 2008; Brodsky et al., 2009, 2010; Neal et al., 2012). The likeability of the expert is a prominent social cue that is readily accessible to jurors. It is considered important because likeability increases juror connection, attention and receptiveness (McGaffey, 1979; Schutz, 1997), thereby fostering perceptions of credibility and merit (Chaiken, 1980; Brodsky et al., 2009; Neal et al., 2012). The importance of likeability for expert credibility assessment is supported by evidence that the Witness Credibility Scale (Brodsky et al., 2010) accounts for ~70% of the observed variance in credibility using just four factors: likeability, confidence, knowledge, and trustworthiness. On its own likeability accounts for ~7% of the variance within this model.

Although likeability is clearly not the sole determinant of jurors' credibility assessments, experimental research further supports the significance of likeability in expert persuasion. For example, Brodsky et al. (2009) presented mock jurors one of two videos of the testimony of an expert who was a licenced clinical psychologist, with an established private practice, 14 years of experience conducting over 100 forensic risk evaluations, and a history of providing expert testimony in over 50 cases. The only difference between the two videos was the level of expert likeability, which was manipulated to be either “low” or “high” using verbal and non-verbal cues such as smiling, body language and deferential speech. The results showed that the likeable expert was rated as more credible and trustworthy than the less likeable expert. Thus, the more likeable expert was more persuasive than a less likeable expert of the same quality.

Adapting the materials used by Brodsky et al. (2009) and Neal et al. (2012) examine the effect of likeability and expert knowledge on perceptions of persuasiveness. In their study, mock jurors watched the testimony of a high or low likeability expert who was either a “high knowledge” experienced clinical psychologist, or a “low knowledge” inexperienced general psychologist. The results showed that the more knowledgeable expert was more credible to jurors than the low knowledge expert. They also found that likeability had a consistent effect, boosting the credibility of both high and low knowledge experts. Taken together, these findings show that likeability does influence perceptions of expert credibility. Yet there is no evidence that a more likeable expert provides evidence that is more scientifically sound, logically coherent, or empirically justified than a less likeable expert (Chaiken, 1980; Petty and Cacioppo, 1986; Greene and Gordan, 2016). Thus, a reliance on likeability may misdirect or misinform juror evaluations and contribute to unjust trial outcomes, especially when a highly likeable expert provides a low-quality opinion. However, it is important to consider the limitations of past research when assessing the potentially negative effects of expert likeability on juror assessments of credibility and persuasiveness.

To-date studies have typically conceptualised and manipulated expert evidence quality in simplistic ways, for example, using abridged trial vignettes, decontextualised expert extracts, and few or basic indicators of quality (e.g., years of experience or prestige of credentials; Petty et al., 1981; Swenson et al., 1984; Guy and Edens, 2003; McAuliff and Kovera, 2008; Brodsky et al., 2009; Neal et al., 2012; Parrott et al., 2015; Salerno et al., 2017). Given these somewhat impoverished materials, it is possible that the information that was available—including about likeability—may gain undue prominence in decision-making. Where peripheral cues are available, they may even “stand in” for useful but unavailable information (Petty and Cacioppo, 1986; Shuman et al., 1994; Sporer et al., 1995; Cooper et al., 1996; Ivković and Hans, 2006; Tenney et al., 2008). For example, there is evidence that likeability is used to make inferences about expert trustworthiness (Neal et al., 2012). Thus, it remains unclear how likeability may impact jurors' assessments of credibility and persuasiveness, when more realistic indicators of expertise are available to inform decision-making.

Another related limitation is the tendency to conflate expert evidence quality and likeability in experimental manipulations. For example, in previous studies, *likeability* manipulations also altered aspects of the evidence quality. Specifically, modest statements that acknowledge limited certainty and the potential for error used in studies such as Brodsky et al. (2009) and Neal et al. (2012) are generally considered to be higher quality than overstated conclusions that fail to acknowledge uncertainty (Koehler, 2012; Edmond et al., 2016). Thus, the influence of likeability on judgments of credibility might not have been entirely attributable to likeability, but rather, may partially be a response to differences in evidence quality. Consequently, it is unclear how influential peripheral cues such as likability are to credibility judgements when they are made in more realistic contexts where expert opinion quality is operationalised in more subtle and realistic ways.

Recent attempts to address this gap in the persuasion literature have used richer representations of expert opinion quality. Martire et al. (2020) operationalised expert opinion quality using the Expert Persuasion Expectancy (ExPEx) Framework. The ExPEx Framework specifies eight attributes that are logically relevant to the quality of an expert opinion: foundation, field, specialty, ability, opinion, support, consistency, and trustworthiness. *Foundation* refers to the empirical validity and reliability of the field in which the expert is opining (e.g., the discipline's error rate). *Field* relates to expert's training, study, and experience in an area generally relevant to their opinion (e.g., clinical psychology training). *Specialty* concerns whether the testifying expert has training, study or experience that is specifically relevant to the assertions they are making (e.g., risk assessment training). *Ability* relates to the expert's track record and their ability to form accurate and reliable opinions (e.g., personal proficiency). *Opinion* concerns the substantive opinion or judgment conveyed by the expert, its clarity, and the acknowledgement of limitations. *Support* concerns the presence and quality of evidence underpinning the opinion (e.g., the results of psychometric testing). *Consistency* relates to the level of agreement amongst different suitable experts. *Trustworthiness* refers to the experts' conscientiousness, objectivity, and honesty.

When information about all ExPEx attributes was available to jury-eligible respondents, participants were more persuaded by objectively high- compared to low-quality forensic gait expert evidence (Martire et al., 2020). Jurors were also particularly influenced by information about the experts' track record (ability), their impartiality (trustworthiness), and the acceptability of their conclusion to other experts (consistency). However, the nuanced operationalisation of expert evidence quality used in this research did not extend to the use of realistic trial materials. Participants were merely presented an eight-statement description of the expert and their opinion and were not given any information about peripheral cues such as likeability. Thus, the influence of likeability on the assessment of expert evidence quality, especially in information-rich decision scenarios, remains unknown. Our research addresses this gap.

Across two studies, jury-eligible participants rated the persuasiveness of an expert opinion and provided a sentencing decision in a Capitol murder case after viewing and/or reading ExPEx-enriched high- or low-quality expert testimony. The materials were adapted from Neal et al. (2012) and Parrott et al. (2015) and included versions of the testimony from a high- or low-likeability expert (Study 1) with a neutral likeability control (Study 2).

In line previous research using the ExPEx framework, we expect that jurors will regard higher quality expert evidence as more persuasive than lower quality evidence, and that sentencing decision will be affected by evidence quality. We also expect that persuasiveness ratings will predict sentencing decisions. In addition, if as previously observed, likeability does influence perceptions of expert credibility and persuasiveness, then we would expect to find that more likeable experts are more persuasive than less likeable experts irrespective of evidence quality. However, if the previous effects of likeability were a result of the simplistic or confounded experimental materials rather than the persuasiveness of likeability *per se*, then we would not expect an effect of likeability because jurors will instead rely on the numerous valid quality indicators available in the trial scenario. Main effects of both quality and likeability, and any interactions between quality and likeability, would suggest that both likeability and indicators of evidence quality affect the persuasiveness of an expert opinion and/or sentencing decisions.

## Study 1

### Method

#### Design

Study 1 used a two (expert evidence quality: low, high) × two (likeability: low, high) between-subjects factorial design. Expert evidence quality was operationalised using either eight “high-quality” or eight “low-quality” ExPEx attributes. Low-vs. high-likeability was operationalised using the trial materials and verbal components from Neal et al. (2012). The primary dependent variables were persuasiveness rating and sentencing decision. Persuasiveness was measured by averaging ratings of expert credibility, evidence value and evidence weight (all rated from 0 to 100). Sentencing decision was a binary choice between life in prison or death sentence per Neal et al. (2012). This study was pre-registered (AsPredicted#: 65017) and materials, data and analyses are available at [link for blind review to be updated if accepted: https://osf.io/yfgke/].

#### Participants

Participants were recruited from Amazon Mechanical Turk (MTurk). All participants resided in the United States and were aged 18 years or older. To maximise data quality, participation was limited to those who had not been involved in our similar studies and who had a 99% MTurk approval rating. Participants also completed attention checks and a reCAPTCHA to exclude non-human respondents (Von Ahn et al., 2008). Two-hundred and forty participants were recruited and were compensated US$2.00 for their time. Participants who either failed the age check, were ineligible to serve on a jury or failed the attention checks (*n* =22), were excluded from the final sample per our pre-registered exclusion criteria. The final sample consisted of 218 jury-eligible participants randomly allocated to condition as follows: high-quality, high-likeability: *n* = 55: high-quality, low-likability: *n* = 57; low-quality, high-likeability: *n* = 50; low-quality, low-likeability: *n* = 56.

#### Materials and Measures

##### Trial Materials

The trial materials used in this study were adapted from Neal et al. (2012) with the permission of the author. Departures from the original materials and procedures are specified below.

##### Pre-trial Instructions

Participants read written jury instructions indicating that the defendant had been found guilty of first-degree murder and that they were to return either a sentence of life in prison, or death, based on whether it could be shown “*beyond a reasonable doubt that there is a probability that the defendant would commit criminal acts of violence that would constitute a continuing danger in society*” (Neal et al., 2012). This jury instruction was adapted from the Texas Criminal Procedure Code, Article 370.071b-f (1985) by Krauss and Sales (2001).

##### Expert Evidence

The expert evidence transcript used by Neal et al. (2012) was based on an actual jury sentencing proceeding and portrayed the examination-in-chief and cross-examination of a forensic psychologist testifying about the likelihood that a convicted murderer would commit future violence (Krauss and Sales, 2001). The expert provided inculpatory evidence, ultimately stating that there is a “*high probability that he will commit future acts of dangerousness”* (Neal et al., 2012).

Participants were presented the original examination-in-chief and cross-examination of the expert used by Neal et al. (2012) without modifications. This transcript contained information about the experts' educational credentials, experience, method for conducting violence risk assessment, and their opinion about the defendants' future risk of violence. This information related to the field, specialty, and support attributes in the ExPEx framework, which together formed the manipulation of expert “knowledge” (see Evidence Quality Manipulation for further detail). Ability, foundation and opinion were also addressed though in a limited way. Specifically, in all conditions, the expert had ultimately concluded that the defendant posed a “*continuing danger to society”* and that despite research showing clinical psychologists can be inaccurate, as far they knew, they had “*never been wrong”* in their evaluations.

To ensure that there was information available about all ExPEx attributes, a three-page ‘re-examination’ was added to enrich the trial transcript. In this supplementary material participants were told that the prosecution and defence have recalled the expert for further testimony, were reminded of the jury instructions before reading the three new pages of written testimony. During the re-direct and cross-examination, the expert provided additional detail about their educational credentials, experience, methodology, and clarified their opinion. They also provided new information about the scientific basis for risk assessment (foundation), their own proficiency conducting risk assessments (ability), whether other experts agreed with their conclusions (consistency), and their track record working for the defence and prosecution (trustworthiness).

**Evidence Quality Manipulation**. All eight ExPEx attributes were manipulated in the transcript to produce either a high- or low-quality opinion (see OSF for evidence quality manipulations).

In the high-quality condition, participants read the materials developed by Neal et al. (2012) presenting the testimony of a clinical psychologist, educated at Yale, with a PhD, who was a Board-certified Forensic Psychologist with several academic publications in forensic risk assessment (field and specialty). The expert had 14 years of specialist training and experience in dangerousness and violence risk assessment and had used multiple clinical interviews totaling 15 h with the defendant to assess risk utilising the Violence Risk Assessment Guide (specialty and support). In the enriched re-examination, participants were also given information that the expert was highly proficient in conducting violence risk assessments, with an average performance of 90–94% accuracy (ability), that clinical psychology is a discipline that equips professionals to make accurate risk judgments, and that the V-RAG is an empirically supported and validated assessment method (foundation). The high-quality condition also read that the clinical psychologist managed the potential for bias in their opinion, had testified equally for the prosecution and defence, and did not know the defendant previously (trustworthiness). They also acknowledged the limits of their conclusion by suggesting that even experts do not always have perfect judgment and that risk assessments are not 100% accurate (opinion). The clinical psychologists' opinion was based on collateral information, interview and addressed the relevant risk factors (support). The opinion was also verified by independent experts in the same specialist field (consistency).

By contrast, adopting Neal et al. (2012) manipulations, participants in the low-quality condition read the testimony of a non-specialist psychologist, with 2 years of experience as a psychotherapist in private practice, who did not provide their educational credentials (field), had no specialisation or experience in violence risk assessment (specialty) and had only completed a 30-min interview with the defendant before using the V-RAG (support). In the enriched re-examination, participants also learned that the psychotherapist had not had their risk assessment performance tested but nevertheless reported they were highly proficient (ability), relied on unvalidated clinical judgment and modified the VRAG to assess risk (foundation and support). Those in the low-quality condition also learned that the psychotherapist had worked mostly for the prosecution, had known the defendant previously, and had only considered information they considered relevant in their personal opinion (trustworthiness). They communicated no uncertainty or limitations around their conclusions when re-clarifying their expert judgment (opinion). The psychotherapist's opinion was based only on information from the 30-min interview, did not refer to empirical literature (support), and was verified by a law enforcement official who was also working on the same case rather than an expert in risk assessment (consistency).

**Likeability Manipulation**. Likeability was manipulated in the original materials using verbal cues (Neal et al., 2012; see OSF for likeability manipulations). The same high and low likeability manipulations were applied throughout the enriched re-examination transcript to ensure consistency throughout the scenario. These likeability manipulations have been shown to be successful at differentiating an expert high in likeability from an expert low in likeability (Brodsky et al., 2009; Neal et al., 2012).

In the high-likeability conditions, participants read a version of the expert who used terms such as “we” or “us” when referring to themselves or others, used informal speech (e.g., referring to an individual by name), was genuine, humble and deferential (e.g., commended the work of others), showed considerate and respectful disagreement, agreeableness to requests and questions (e.g., stating “of course” when asked to repeat something), and had a pleasant and friendly interpersonal style.

In the low-likeability conditions, participants read a version of the expert who used individualistic pronouns (i.e., I, me), was disingenuous, arrogant, and non-deferential (e.g., displayed superiority relative to others, was self-complimenting), showed aggressive contradiction and disagreement, disagreeableness in response to requests, and questions (e.g., pointing out repetitiveness and labelling questions as redundant), and had an unfriendly and condescending interpersonal style.

##### Primary Dependant Measures

**Persuasiveness**. The persuasiveness measure comprised three questions. Participants rated the credibility of the expert (“*how credible is Dr. Morgan Hoffman?”*) from 0 “not at all” to 100 “definitely credible,” the value of the expert's evidence (“*how valuable was Dr. Morgan Hoffman's testimony?*”) from 0 “not at all” to 100 “definitely valuable,” and the weight of the expert's evidence (“*how much weight do you give to Dr. Morgan Hoffman's testimony?”*) on a scale from 0 “none at all” to 100 “the most possible.” Question order was randomised. These items have been previously found to be highly correlated (all *r'*s > 0.847) and have high internal consistency (Cronbach's α = 0.954; Martire et al., 2020).

**Sentencing Decision**. Participants were asked “*Considering all the evidence provided to you, what is the sentence you would recommend for the defendant?”* and were required to answer either “I would recommend that the defendant receive a death sentence” or “I would recommend that the defendant receive a sentence of life in prison.”

##### Secondary Measures & Manipulation Checks

###### ExPEx Attribute Ratings.

Eight items were used to assesses decision-makers' perceptions of whether or not the expert opinion had a high-quality foundation, field, specialty, ability, opinion, support, consistency and trustworthiness from 0 “not at all” to 100 “definitely.” Question order was randomised. See Table 1 for verbatim wording and format.

**Table 1:** Expert persuasion expectancy (ExPEx) quality items.

**ExPEx Attribute**	**Question**
Foundation	Does training, study, and experience in clinical psychology **support assertions** that a *defendant will commit a violent offence and pose a danger to society*?
Field	Does Dr. Morgan Hoffman have **training, study, and/or experience** in *clinical* psychology?
Specialty	Does Dr. Morgan Hoffman **have training, study and/or experience specific** to making assertions that *defendant will commit a violent offence and pose a danger to society*?
Ability	Does Dr. Morgan Hoffman make assertions that the *defendant will commit a violent offence and pose a danger to society* **accurately and reliably**?
Opinion	Did Dr. Morgan Hoffman convey their assertion that the *defendant will commit a violent offence and pose a danger to society* **clearly**, and with **necessary qualifications/limitations**?
Support	Did Dr. Morgan Hoffman **rely on evidence** when forming their assertion that the *defendant will commit a violent offence and pose a danger to society*?
Consistency	Is Dr. Morgan Hoffman's assertion that *defendant will commit a violent offence and pose a danger to society*? **consistent** with what other experts in clinical psychology would assert?
Trustworthiness	Do you believe that Dr. Morgan Hoffman is **fair, impartial**, and **objective**?

*The bolded writing reflects the definitional component of the ExPEx attributes and the italicised writing reflects the key statement (i.e., the expert's conclusion) from which the ExPEx attribute is being rated in accordance with*.

###### Witness Credibility Scale.

The Witness Credibility Scale (WCS) is a 20-item measure assessing expert credibility (Brodsky et al., 2010). Each item contains bipolar adjectives on a 10-point Likert scale [e.g., *not confident* (1) to *confident* (10)]. The presentation of the items was randomised. The highest possible score for overall credibility is 200, with higher scores indicating higher credibility ratings. The WCS also yields a sub-scale score for four credibility domains: knowledge, trustworthiness, confidence, and likeability. The highest possible score for each domain is 50, with a higher score indicating higher rating in a domain. The WCS has good validity and reliability—it can successfully differentiate between expert displaying varying levels of the four sub-domains (Brodsky et al., 2010). The WCS was included to provide an embedded measure of expert likeability. Participants were asked to rate the expert on the following bipolar adjectives: *unfriendly* (1) to *friendly* (10); *unkind* (1) to *kind* (10); *disrespectful* (1) to *respectful* (10); *ill-mannered* (1) to *well-mannered* (10) and *unpleasant* (1) to *pleasant* (10). Collectively, these items produced the WCS-Likeability subscale.

###### Agreement.

Participants were asked “*If Dr. Hoffman reported that the defendant will commit a violent offence and pose a danger to society, would you agree with that opinion?”* and were required to answer either “yes” or “no.” Analyses involving agreement are available on OSF.

###### Likeability.

Participants were asked to “*rate how likeable Dr. Morgan Hoffman is to you, with zero being ‘not at all likeable’ and ten being ‘extremely likeable’.”*

###### Expert Testimony Comprehension.

Comprehension of the expert evidence was measured using 6 multiple-choice items to assess engagement with the testimony and understanding of its substantive content. Higher comprehension scores (out of 6) indicated greater recall and comprehension of the expert testimony. Analyses involving comprehension are available on OSF.

###### Demographic Information.

Participants were asked to provide information about their age, gender, education level, cultural background, English proficiency, religiosity, political orientation, views of the death penalty, experience and familiarity with the expert's discipline and jury eligibility and experience.

#### Procedure

This study was approved by the UNSW Human Advisory Ethics Panel C—Behavioural Sciences (Approval #3308) and pre-registered. The study was advertised on MTurk and completed by participants online in Qualtrics. Before commencing the study, participants were asked to provide informed consent, complete age eligibility and reCAPTCHA, before random allocation to condition. Participants read the instructions and the version of the expert testimony transcript as determined by allocation to condition. Next, participants completed the ExPEx, WCS and likeability measure, in randomised order; completed the persuasiveness measure, and made their sentencing decision. Finally, participants completed the comprehension and demographic items. At the conclusion of the study, participants were given a completion code, were debriefed, and thanked. The average study completion time was 23.5 min.

### Results

#### Participant Demographics

Participants were aged between 18 and 71 years (M = 39.36, SD =12.34) and 48.6% were male. Most participants reported that their highest level of completed education was college/university (54.1%) or a Masters degree (27.5%). Most identified as White/Caucasian (71.6%), followed by Asian (10.6%), African American (8.3%), and Hispanic (5%). Almost all participants (95.9%) were native English speakers.

About half of participants (53.2%) considered themselves more than “moderately” religious (on a 10-point scale from “not at all” to “very” religious). The largest proportion of participants (45.5%) rated themselves as conservative (on a 10-point scale from “very liberal” to “very conservative;” 42.2% were liberal; and 12.4% were neutral). The largest proportion of participants (47.7%) were in favour of the death penalty (on a 10-point scale from “strongly opposed” to “strongly in favor;” 45% were not in favour; 7.3% were neutral). A majority (61.5%) were unfamiliar with dangerousness and violence risk assessments (“none” to “some” familiarity), but approximately half (52.8%) reported being familiar with psychology/clinical psychology (from “some” to “extensive” familiarity). More than half of the sample (56.9%) had been called for jury duty; 46.8% of these participants had served on a jury, and 7.3% (*n* = 9) had served on a murder trial.

#### Manipulation Checks

All assumptions were tested before conducting the planned analyses. The analytic approach reported here either satisfies the relevant assumptions or is robust to violations.

##### Evidence Quality

A two-way (Pillai's Trace) MANOVA was conducted comparing the ratings of each of the eight ExPEx expert attributes between the low- and high-quality expert evidence conditions. There was a significant main effect of expert evidence quality overall [F_(8,207)_ = 11.9, *p* < 0.001, ηp^2^ = 0.315] and for each attribute [all $F^{\prime }s_{\left(1,\;214\right)}$ ≥ 25.83, all *p's* < 0.001, all ηp^2^ ≥ 0.108] such that, on average, participants in the high-quality condition rated each ExPEx attribute as higher quality than those in the low-quality condition (see Table 2).

**Table 2:** Table of marginal means and inferential statistics for expert persuasion expectancy (ExPEx) attributes by evidence quality condition.

	**ExPEx Attribute Rating**						
**ExPEx Attribute**	**High-Quality Mean (SE)**	**95% CI**	**Low-Quality Mean (SE)**	**95% CI**	***F***	***p***	**η^2^**
Foundation	76.62 (2.18)	72.32, 80.92	60.70 (2.25)	56.28, 65.13	25.83	<0.001	0.108
Field	92.64 (1.82)	89.04, 96.23	72.86 (1.88)	69.17, 76.56	57.10	<0.001	0.211
Specialty	88.16 (1.99)	84.24, 92.09	63.08 (2.05)	59.04, 67.12	77.04	<0.001	0.265
Ability	78.93 (2.34)	74.32, 83.53	57.24 (2.41)	52.50, 61.98	41.82	<0.001	0.163
Opinion	82.87 (2.39)	78.16, 87.59	64.33 (2.46)	59.47, 69.18	29.18	<0.001	0.120
Support	76.48 (2.34)	71.87, 81.10	58.22 (2.41)	53.47, 62.97	29.56	<0.001	0.121
Consistent	78.74 (2.08)	74.64, 82.84	57.97 (2.14)	53.74, 62.19	48.40	<0.001	0.184
Trustworthy	73.07 (2.61)	67.93, 78.21	51.05 (2.69)	45.76, 56.34	34.59	<0.001	0.139

##### Likeability

Independent samples Welch *t*-tests showed a significant difference between high- and low-likeability experts on the WCS-likeability sub-scale score [*t*_(163.79)_ = −10.99, 95% CI (−20.78, −14.45), *p* < 0.001]. On average participants in the high-likeability condition rated the expert 39.3 out of 50 (SD = 7.3) compared to 21.7 out of 50 (SD = 15.3) in the low-likeability condition.

The single item rating subjective likeability was strongly and positively correlated with the WCS-Likeability subscale score (*r* = 0.921, *p* < 0.001). Accordingly, we report all subsequent analyses using the validated WCS-Likeability scores rather than the single likeability item.

#### Persuasiveness Ratings

Consistent with Martire et al. (2020), ratings of expert credibility, value and weight were all strongly and positively correlated (*r*_credibility/weight_ = 0.905; *r*_credibility/value_ = 0.894; *r*_value/weight_ = 0.913, all *p's* < 0.001), and had high internal consistency (Cronbach's α = 0.966), so were combined into a single measure of persuasiveness.

#### Effect of Expert Evidence Quality and Likeability on Persuasiveness

**Figure 1:**
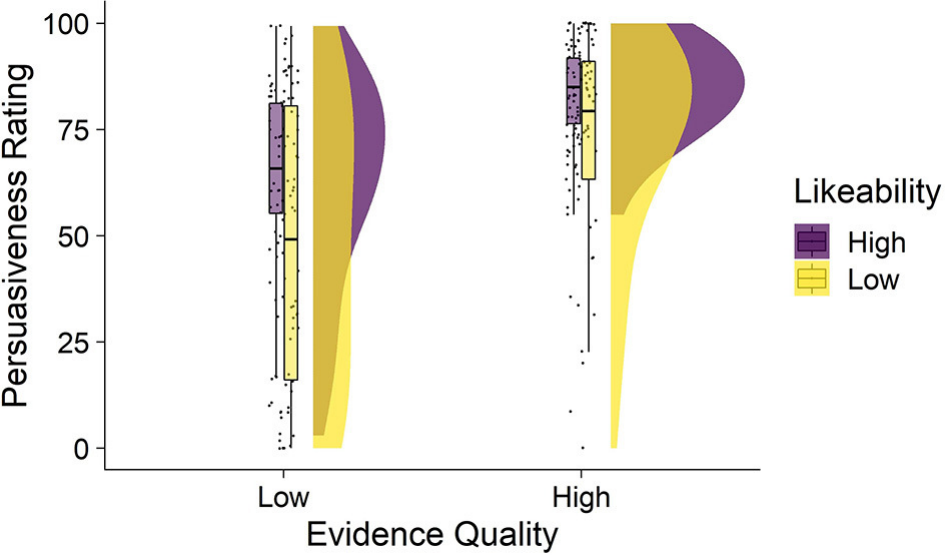
Persuasiveness as a function of expert evidence quality and likeability (Study 1). Figure depicts four raincloud plots showing the distribution of persuasiveness ratings observed in each condition. From left to right, each raincloud plot shows the: jittered individual data points, box-and-whisker plots (middle bar within the box is the median, the box represents the interquartile range of persuasiveness ratings, and the whiskers represent persuasiveness ratings no further than 1.5 × the interquartile range), and the distributions showing the frequency of persuasiveness ratings. Mean persuasiveness ratings differed by evidence quality and likeability conditions.

Average persuasiveness ratings by condition are shown in Figure 1. The mean persuasiveness ratings by condition were: high-quality, high-likeability *M* = 84.5 (SD = 11): high-quality, low-likability *M* = 72.8 (SD = 24.2); low-quality, high-likeability *M* = 62.7 (SD= 24.4); low-quality, low-likeability *M* = 47.9 (SD = 32). A two-way ANOVA showed a significant main effect of evidence quality [*F*_(1, 214)_ = 50.76, *p* < 0.001, ηp^2^ = 0.192] and a significant main effect of likeability [*F*_(1, 214)_ = 16.39, *p* < 0.001, ηp^2^ = 0.071]. The high-quality expert was significantly more persuasive than the low-quality expert. The high-likeability expert was also more persuasive than the low-likeability expert. There was no significant interaction between evidence quality and likeability indicating that the effect of likeability was consistent for high- and low-quality evidence [*F*_(1, 214)_ = 0.234, *p* = 0.629, η*p*^2^ = 0.001].

Multiple regressions were conducted to examine whether continuous subjective ratings of the eight ExPEx attributes and WCS-likeability predicted persuasiveness ratings. The overall model was significant [*F*_(9, 208)_ = 111.06, *p* < 0.001] and accounted for 82% of the variance in persuasiveness ratings (adjusted *R*^2^ = 0.82). Ratings of trustworthiness, consistency, support, ability, and specialty were all significant independent predictors (all *p'*s ≤ 0.022), while foundation, field, opinion, and likeability were not (all *p'*s ≥ 0.054; see Table 3). For example, holding all else constant, a one unit increase in perceptions of the trustworthiness of the expert was associated with a 0.347 unit increase in persuasiveness ratings.

**Table 3:** Multiple regression predicting persuasiveness from continuous expert persuasiveness expectancy (ExPEx) ratings and witness credibility score (WCS) for likeability.

		**95% CI for** ***B***						
	***B***	**Lower**	**Upper**	**SE *B***	**β**	***p***	***R* ^2^**	**Adj. *R*^2^**
Model						<0.001	0.828	0.82
Foundation	−0.048	−0.143	0.047	0.048	−0.042	0.324		
Field	0.000	−0.097	0.098	0.05	0.000	0.993		
Specialty	0.18^***^	0.071	0.288	0.055	0.161^***^	0.001		
Ability	0.237^***^	0.141	0.334	0.049	0.232^***^	<0.001		
Opinion	0.021	−0.063	0.105	0.042	0.021	0.619		
Support	0.148^***^	0.069	0.226	0.04	0.142^***^	<0.001		
Consistent	0.119^*^	0.017	0.221	0.052	0.107^*^	0.022		
Q0Trustworthy	0.347^***^	0.254	0.44	0.047	0.395^***^	<0.001		
WCS-Likeability	0.15	−0.002	0.303	0.077	0.081	0.054		

*B, unstandardised regression coefficient; CI, confidence interval; SE B, standard error of the coefficient; β, standardised coefficient*.

* *p < 0.05*,

^**^p < 0.01,

*** *p ≤ 0.001*.

#### Relationship Between Persuasiveness and Sentencing Decision

A binominal logistic regression was used to examine the relationship between persuasiveness and sentencing decision. The overall model was a good fit and significant [$\chi_{\left(1\right)}^{2}$ = 56.14, *p* < 0.001]. Persuasiveness accounted for 31.7% of the variance in sentencing decision [Nagelkerke *R*^2^ = 0.317; Wald $\chi_{\left(1\right)}^{2}$ = 29.79, *p* < 0.001], with a one unit increase in persuasiveness increasing the odds of the decision-maker choosing a death sentence by 1.063 (Exp *B*).

#### Effect of Expert Evidence Quality and Likeability on Sentencing Decision

The proportion of participants giving death sentences by condition is shown in Table 4. A binominal logistic regression was used to predict sentencing decision from expert quality condition, likeability condition, and their interaction. The overall model was a good fit but not significant [$\chi_{\left(3\right)}^{2}$ = 6.84, *p* = 0.077] and accounted for only 4.3% of the variance in sentencing decision (Nagelkerke *R*^2^ = 0.043). Neither expert evidence quality, likeability, nor their interaction were significant independent predictors of sentencing decision (all *p's* ≥ 0.158; see Table 5).

**Table 4:** Proportion of participants selecting death sentence by evidence quality and likeability condition.

**Likeability**	**Low-Quality Evidence %**	**High-Quality Evidence %**
Low	26.8	43.9
High	22	34.5

**Table 5:** Logistic regression predicting sentencing decision from evidence quality condition, likeability condition, and their interaction.

	***B***	**SE**	**Wald**	***df***	***p***	**Odds Ratio**	**95% CI for Odds Ratio**	
							**Lower**	**Upper**
Evidence quality	−0.63	0.44	1.99	1	0.158	0.53	0.22	1.28
Likeability	0.39	0.39	1.01	1	0.314	1.48	0.69	3.18
Evidence quality^*^ likeability	−0.13	0.6	0.05	1	0.826	0.88	0.27	2.84

*Life in prison was coded as zero and death was coded as one. B, unstandardised regression coefficient; SE B, standard error of the coefficient; CI, confidence interval*.

* *p < 0.05*,

^**^p < 0.01, ^***^p ≤ 0.001.

Another binominal logistic regression conducted to examine whether subjective continuous ExPEx ratings and WCS-likeability scores predicted sentencing decision. The overall model was a good fit and was significant [$\chi_{\left(9\right)}^{2}$ = 53.35, *p* < 0.001], accounting for 30.4% of the variance in sentencing decision (Nagelkerke *R*^2^ = 0.304). Ratings of foundation (*p* = 0.022) and trustworthiness (*p* = 0.004) uniquely predicted sentencing decision, while the remaining ExPEx attributes and likeability scores did not (all *p'*s ≥ 0.104; see Table 6).

**Table 6:** Logistic regression predicting sentencing decision from continuous expert persuasion expectancy (ExPEx) ratings and witness credibility score (WCS) for likeability.

	***B***	**SE**	**Wald**	***df***	***p***	**Odds Ratio**	**95% CI for Odds Ratio**	
							**Lower**	**Upper**
Foundation^*^	0.028^*^	0.012	5.25	1	0.022	1.03	1.00	1.05
Field	−0.009	0.012	0.6	1	0.439	0.99	0.97	1.01
Specialty	−0.004	0.014	0.095	1	0.758	0.996	0.97	1.02
Ability	0.002	0.013	0.03	1	0.869	1.00	0.98	1.03
Opinion	0.016	0.012	1.83	1	0.177	1.02	0.99	1.04
Support	<0.001	0.008	0.001	1	0.974	1	0.98	1.02
Consistent	−0.006	0.012	0.293	1	0.588	0.99	0.97	1.02
Trustworthy	0.034^**^	0.012	8.46	1	0.004	1.03	1.01	1.06
WCS-Likeability	−0.027	0.017	2.64	1	0.104	0.97	0.94	1.01

*Life in prison was coded as zero and death was coded as one. B, unstandardised regression coefficient; SE B, standard error of the coefficient; CI, confidence interval*.

* *p < 0.05*,

** *p < 0.01*,

^***^p ≤ 0.001.

### Discussion

Study 1 examined whether expert quality and likeability affected jury-eligible participants' perceptions of expert persuasiveness and sentencing decisions. We found that participants' perceptions of persuasiveness were significantly affected by evidence quality and expert likeability whereby higher quality and higher likeability experts were more persuasive than lower quality and lower likeability experts. However, there was no interaction between evidence quality and likeability. We also found that subjective perceptions of the eight ExPEx attributes and likeability together accounted for ~80% of the variance in persuasiveness scores, which demonstrates these attributes have strong predictive power.

These results suggest that evidence quality impacts understanding, but persuasiveness is determined by both the underlying quality of the evidence, and superficial aspects of the experts' interpersonal style. Our results also suggest that previously observed effects of likeability on perceptions of credibility or persuasiveness were not merely an artefact of simplified evidence quality materials and manipulations. The expert evidence presented in this study was detailed and included extensive information about the quality of the opinion, yet the effect of likeability persisted and appeared to provide a boost to the persuasiveness of both lower and higher quality evidence. Thus, concerns about juror reliance on peripheral information in their decision-making remain.

However, it is important to note that neither likeability nor quality affected sentencing decisions in the same way that they affected persuasiveness. There were no significant associations between evidence quality or likeability conditions on sentencing outcomes. Continuous subjective likeability ratings also did not predict sentencing outcome, but perceptions of expert trustworthiness and foundation did. Thus, although likeability affected perceptions of persuasiveness, and persuasiveness affected sentencing outcomes, likeability did not directly affect the final sentencing outcome. This was not the case for evidence quality—elements of which remained influential for both evidence evaluation and sentencing decisions. Taken together, this suggests that lay decision-makers consider elements of expert evidence quality more so than peripheral likeability information when making their sentencing decisions.

These results raise further questions that should be explored. First, it is important to establish whether these effects are reliable by attempting to replicate the results. It is also important to consider whether our results are generalisable, especially given the lower ecological validity of trial transcript studies. Perceptions of likeability are strongly affected by non-verbal cues such as smiling, nodding, eye contact and open posture (Kleinke, 1986; Leathers, 1997; Gladstone and Parker, 2002). These cues were not available in our materials. Thus, it is important to examine whether the effects of expert likeability are replicated when more realistic video manipulations of likeability are used. Finally, we were interested to inform our general understanding of the relationship between likeability and persuasion by considering likeability's directional impact on persuasiveness. It is unclear whether being likeable *increases* persuasiveness, or if it is being *dis*liked that *decreases* persuasiveness, or both. Study 2 is designed to tease apart these possibilities.

## Study 2

### Method

#### Design

Study 2 used a 2 (expert evidence quality: high, low) × 3 (likeability: neutral, low, high) between-subjects factorial design. The dependent variables and evidence quality manipulations were the same as Study 1. Details about the likeability manipulations are described below. Study 2 was pre-registered (AsPredicted#: 39310) and material, data and analyses are available at [blind link to OSF: https://osf.io/yfgke/].

#### Participants

Participants were recruited using two methods: (1) online via MTurk, with the same quality assurance methods as in Study 1, and (2) via the UNSW first-year psychology undergraduate student pool. Research suggests that online and undergraduate participant samples are generally comparable and there is little evidence of significant differences in the decisions made between student and non-student samples in mock jury decision-making research (Bornstein and Greene, 2011; Buhrmester et al., 2011).

MTurk participants were compensated $US4.00 for their time, while the first-year psychology students received course credit. Participants who did not consent, failed the audio check, failed the attention checks, or were ineligible to serve on a jury (*n* = 110) were excluded from the final sample as per pre-registered exclusion criteria. The final sample consisted of 238 jury-eligible participants (164 from MTurk and 74 from the undergraduate pool), randomly allocated to condition as follows: high-quality, neutral likeability *n* = 39; high-quality, high-likeability *n* = 44: high-quality, low-likability *n* = 37; low-quality, neutral likeability *n* = 37; low-quality, high-likeability *n* = 44; low-quality, low-likeability *n* = 37.

#### Materials and Measures

The same trial scenario, expert evidence quality manipulations, and manipulation checks from Study 1 were used in Study 2 except as described below. Changes were made to the likeability manipulation to incorporate the new neutral likeability condition.

##### Expert Quality and Likeability

To increase realism of the likeability manipulation the transcript of the examination-in-chief and cross-examination of the high- and low-quality and high- and low-likeability expert evidence was replaced with video reenactments also produced by Neal et al. (2012). In these videos participants saw a White middle-aged male providing testimony from a courtroom, with a US flag in the background. The videos were between 4.5 and 6 min long and displayed the same actor to control for between-person characteristics (i.e., attractiveness). In addition to the verbal likeability cues from Study 1, participants in the high likeability condition saw an expert who showed moderate levels of smiling, consistent eye contact, open body language and a modest presentation style. Those in the low likeability condition saw an expert who did not smile, had inconsistent eye contact, closed body language and a conceited presentation style. These videos were followed by the same ExPEx enriched transcript developed for Study 1.

The materials for the new neutral likeability condition were presented in transcript format to minimise all visual likeability cues (e.g., smiling, eye contact). Participants in this condition read a transcribed version of the same examination-in-chief and cross-examination video. The transcript was developed by Parrott et al. (2015) and removed or neutralised the likeability cues contained in the original Neal et al. (2012) materials. For example, phrases such as “I take this responsibility very seriously,” “of course” or “feeble-minded people think they know everything” were removed leaving only the essential substantive content. Participants also read a neutral version of the enriched transcript stripped of the likeability cues added for Study 1.

The manipulation checks, dependent and secondary measures were the same as in Study 1, except for the comprehension measures which were modified to reflect the testimony as presented in Study 2. Participants also completed the Scientific Reasoning Scale (Drummond and Fischhoff, 2017) and Need for Cognition measure (Cacioppo and Petty, 1982), however analysis of these data was beyond the scope of this study and so is not reported here.

#### Procedure

This study was approved by the UNSW Human Advisory Ethics Panel C—Behavioural Sciences (Approval #3308) and pre-registered. The study was advertised on MTurk and undergraduate recruitment system and was completed online by all participants in Qualtrics. Before commencing the study, participants were asked to provide informed consent, complete age eligibility, a reCAPTCHA and were randomly allocated to condition. Participants read the study instructions, completed an audiovisual check, and watched/read the version of the expert testimony as determined by quality and likeability condition. Next, participants completed the ExPEx, WCS and likeability measures, in randomised order. Participants then completed the persuasiveness measures and made their sentencing decision. Finally, all participants completed the comprehension items, attention checks, secondary measures, and demographic questions. At the conclusion of the study, participants were given a completion code, were debriefed, and thanked. The average study completion time was 41 min.

### Results

Before conducting the planned analyses, the assumptions were tested for all the statistical procedures employed and were robust. Initial analyses were conducted separately for undergraduate and MTurk participants. The results for these two groups varied in minor ways due to the disparate sample sizes but were broadly consistent, so we present the combined analysis here. Data and the primary persuasion analysis for each sample are available on OSF.

#### Participant Demographics

Overall, participants were aged between 18 and 72 years (M = 31.8, SD =12.2) and 51.3% were male. Most participants reported that college/university (43.3%) or high/secondary school (37.4%) was their highest level of completed education. Most participants identified as White/Caucasian (69.7%), followed by Asian (13.9%), African American (5.5%), and Other (4.6%). Almost all participants (95.4%) were native English speakers.

About half of participants (52.5%) considered themselves more than “moderately” religious. The largest proportion of participants (45%) rated themselves as conservative (39.9% were liberal; 15.1% were neutral). Just over half of participants (52.1%) were against the death penalty (38.7% were in favour; 9.2% were neutral). About half (50.4%) reported they were unfamiliar with clinical psychology and two-thirds (64.3%) were unfamiliar with dangerousness and violence risk assessment. One-third (32.4%) of the sample had been called up for jury duty; 51.9% of these participants had served on a jury, and 8% (*n* = 3) had served on a murder trial.

#### Manipulation Checks

##### Evidence Quality

A two-way (Pillai's Trace) MANOVA was conducted comparing the ratings of each of the eight ExPEx attributes between the low- and high-quality expert evidence conditions. There was a significant main effect of expert evidence quality overall [*F*_(8,225)_ = 5.46, *p* < 0.001, η*p*^2^ = 0.163] and for each ExPEx attribute (all *F's*_(1, 232)_ ≥ 8.6, all *p's* ≤ 0.004, all ηp^2^ ≥ 0.036) such that, on average, participants in the high-quality expert evidence conditions rated each ExPEx attributes as higher quality compared to those in the low-quality condition (see Table 7).

**Table 7:** Table of marginal means and inferential statistics for expert persuasion expectancy (ExPEx) attributes by evidence quality condition.

	**ExPEx Attribute Rating**						
**ExPEx Attribute**	**High-Quality Mean (SE)**	**95% CI**	**Low-Quality Mean (SE)**	**95% CI**	***F***	***p***	**η^2^**
Foundation	74.79 (2.03)	70.79, 78.78	65.51 (2.05)	61.47, 69.54	10.37	0.001	0.043
Field	88.75 (1.78)	85.25, 92.25	76.85 (1.79)	73.32, 80.38	22.27	<0.001	0.088
Specialty	84.72 (2.04)	80.69, 88.74	66.46 (2.06)	62.40, 70.52	39.59	<0.001	0.146
Ability	75.88 (2.23)	71.48, 80.28	62.23 (2.25)	57.79, 66.66	18.54	<0.001	0.074
Opinion	76.80 (2.24)	72.38, 81.22	62.20 (2.26)	61.74, 70.66	11.06	0.001	0.045
Support	71.66 (2.32)	67.09, 76.22	62.01 (2.34)	57.41, 66.61	8.6	0.004	0.036
Consistent	76.06 (2.07)	71.98, 80.13	62.37 (2.09)	58.26, 66.48	21.71	<0.001	0.086
Trustworthy	73.66 (2.41)	68.91, 78.40	57.37 (2.43)	52.58, 62.15	22.67	<0.001	0.089

##### Likeability

On average participants in the neutral likeability condition rated the expert 38 out of 50 for likeability (SD = 6.8) compared to 40 (SD = 6.7) in the high-likeability condition and 22.5 (SD = 13.5) in the low-likeability condition. A one-way (Welch) ANOVA showed a significant difference in likeability scores by condition [Welch's *F*_(2, 142.321)_ = 51.87, *p* < 0.001]. Follow-up Games-Howell comparisons showed that ratings in the neutral and high-likeability conditions did not differ from each other [M_diff_ high vs. neutral = 2.03, 95% CI (−0.48, 4.53), *p* = 0.138], though likeability was significantly higher in both these conditions than in the low likeability condition [M_diff_ neutral vs. low = 15.5, 95% CI (11.33, 19.67), *p* < 0.001; M_diff_ high vs. low] = 17.53, 95% CI (13.43, 21.63), *p* < 0.001]. Thus, it appeared that adding visual and verbal likeability cues did not significantly increase likeability perceptions beyond the transcript. However, visual, and verbal cues to decrease likeability were effective.

#### Persuasiveness Ratings

Consistent with Study 1, ratings of credibility, weight and value were strongly and positively correlated (*r*_credibility/weight_ = 0.849; *r*_credibility/value_ = 0.879; *r*_value/weight_ = 0.899, all *p's* < 0.001), and had high internal consistency (Cronbach's α = 0.955).

#### Effect of Expert Evidence Quality and Likeability on Persuasiveness

**Figure 2:**
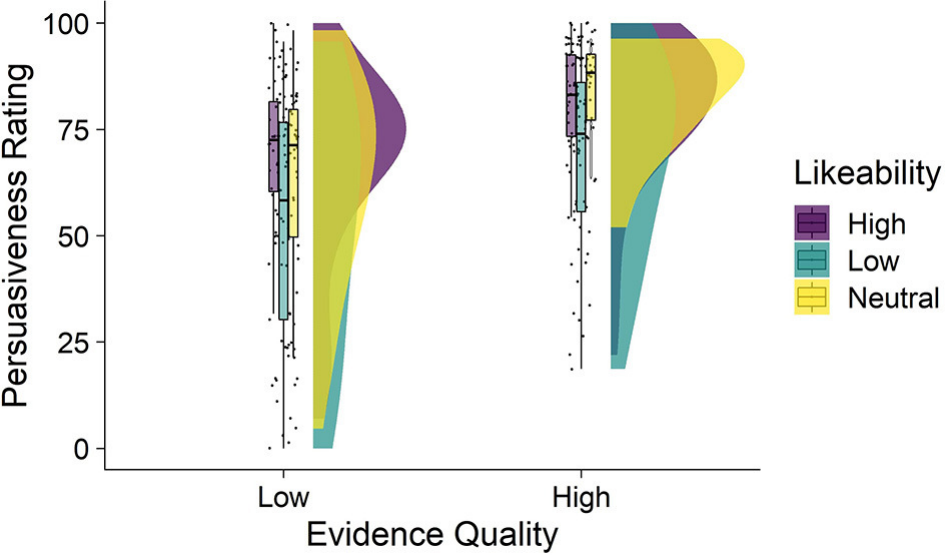
Persuasiveness as a function of expert evidence quality and likeability (Study 2). Figure depicts four raincloud plots showing the distribution of persuasiveness ratings observed in each condition. From left to right, each raincloud plot depicts the: jittered individual data points, box-and-whisker plots (middle bar within the box is the median, the box represents the interquartile range of persuasiveness ratings, and the whiskers represent persuasiveness ratings no further than 1.5 × the interquartile range), and the distributions showing the frequency of persuasiveness ratings. Mean persuasiveness ratings differed by evidence quality and likeability conditions.

Average persuasiveness ratings by condition are shown in Figure 2. The mean persuasiveness ratings by condition were: high-quality, neutral likeability *M* = 83.5 (SD = 11.9); high-quality, high-likeability *M* = 80.6 (SD = 17.2); high-quality, low-likability *M* = 69.1 (SD= 22.4); low-quality, neutral likeability *M* = 62.8 (SD = 23.7); low-quality, high-likeability *M* = 66.9 (SD= 22.7); low-quality, low-likeability *M* = 53.2 (SD = 29).

A two-way ANOVA showed a significant main effect of evidence quality [*F*_(1, 232)_ = 35.58, *p* < 0.001, ηp^2^ = 0.133] whereby higher quality evidence resulted in higher persuasiveness ratings on average compared to lower quality evidence. There was also a significant main effect of likeability [*F*_(2,232)_ = 8.23, *p* < 0.001, ηp^2^ = 0.066]. Follow-up main effects (Tukey HSD) analysis showed that across evidence quality conditions, there was no significant difference in persuasiveness between the high and neutral likeability conditions [M_diff_ high and neutral = 0.28, 95% CI (−7.72, 8.29), *p* = 0.996], however, both the high and neutral conditions resulted in significantly higher persuasiveness ratings than the low-likeability condition [M_diff_ neutral and low = 12.28, 95% CI (3.93, 20.62), *p* = 0.002; M_diff_ high and low = 12.56, 95% CI (4.5, 20.62), *p* = 0.001].There was no significant interaction between evidence quality and likeability [*F*_(2,232)_ = 0.557, *p* = 0.574, ηp^2^ = 0.005] indicating that the effect of likeability was the same across both evidence quality conditions.

Multiple regressions were conducted to examine whether ratings of the eight ExPEx attributes and WCS-likeability predicted persuasiveness ratings. The overall model was significant [*F*_(9,228)_ = 104.74, *p* < 0.001] and accounted for 79.8% of the variance in persuasiveness ratings (adjusted *R*^2^ = 0.798). Ratings of trustworthiness, specialty, opinion, and likeability were significant independent predictors of persuasiveness (all *p'*s ≤ 0.003); foundation, field, ability, support, and consistency were not (all *p'*s ≥ 0.059; see Table 8).

**Table 8:** Multiple regression predicting persuasiveness from continuous expert persuasiveness expectancy (ExPEx) ratings and witness credibility score (WCS) for likeability.

	**CI**_95%_ **for** ***B***							
**Persuasiveness**	***B***	**Lower**	**Upper**	**SE *B***	**β**	***p***	***R^2^***	**Adj. *R*^2^**
Model						<0.001	0.805	0.798
Foundation	0.056	−0.031	0.144	0.044	0.053	0.206		
Field	−0.064	−0.154	0.027	0.046	−0.054	0.166		
Specialty	0.202^***^	0.113	0.291	0.045	0.203^***^	<0.001		
Ability	0.09	−0.004	0.184	0.048	0.097	0.059		
Opinion	0.131^**^	0.048	0.214	0.042	0.14^**^	0.002		
Support	−0.016	−0.092	0.061	0.039	−0.017	0.686		
Consistent	0.066	−0.027	0.158	0.047	0.065	0.162		
Trustworthy	0.383^***^	0.299	0.467	0.042	0.462^***^	<0.001		
WCS-Likeability	0.232^**^	0.08	0.385	0.077	0.119^**^	0.003		

B, unstandardised regression coefficient; CI, confidence interval; SE B, standard error of the coefficient; β, standardised coefficient. ^*^p < 0.05,

** *p < 0.01*,

*** *p ≤ 0.001*.

#### Relationship Between Persuasiveness and Sentencing Decision

The binominal logistic regression testing the relationship between persuasiveness and sentencing decision was a good fit and was significant [$\chi_{\left(1\right)}^{2}$ = 20.72, *p* < 0.001]. Persuasiveness accounted for 12.2% of the variance in sentencing decision [Nagelkerke *R*^2^ = 0.122; Wald $\chi_{\left(1\right)}^{2}$ = 15.44, *p* < 0.001], with a one unit increase in persuasiveness increasing the odds of a death sentence by 1.036 (Exp *B*).

#### Effect of Expert Evidence Quality and Likeability on Sentencing Decision

The proportion of death sentences by condition is shown in Table 9. The binominal logistic regression predicting sentencing decision from expert quality and likeability conditions and their interaction produced a good fit for the data but was not significant [$\chi_{\left(5\right)}^{2}$ = 1.52, *p* = 0.911], accounting for just 0.9% of the variance in sentencing decision (Nagelkerke *R*^2^ = 0.009). Neither quality, likeability, nor their interactions were significant independent predictors of sentencing decision (all *ps* ≥ 0.407; see Table 10).

**Table 9:** Proportion of participants selecting death sentence by evidence quality and likeability condition.

**Likeability**	**Low-Quality Evidence %**	**High-Quality Evidence %**
Neutral	21.6	30.8
Low	27	21.6
High	29.5	27.3

**Table 10:** Logistic regression predicting sentencing decision from evidence quality condition, likeability condition, and their interaction.

	***B***	**SE**	**Wald**	***df***	***p***	**Odds Ratio**	**95% CI for Odds Ratio**	
							**Lower**	**Upper**
Expert evidence quality	0.11	0.47	0.06	1	0.813	1.12	0.44	2.83
Likeability (1)	0.17	0.49	0.12	1	0.726	1.19	0.46	3.07
Likeability (2)	−0.31	0.52	0.34	1	0.558	0.74	0.26	2.05
Expert evidence quality (1) ^*^ likeability (1)	−0.59	0.71	0.69	1	0.41	0.56	0.14	2.23
Expert evidence quality (1) ^*^ likeability (2)	0.18	0.72	0.06	1	0.8	1.2	0.29	4.94

*Life in prison was coded as zero and death was coded as one. B, unstandardised regression coefficient; SE B, standard error of the coefficient; CI, confidence interval*.

* *p < 0.05*,

^**^p < 0.01, ^***^p ≤ 0.001.

The binominal logistic regression testing whether continuous ratings of the eight ExPEx attributes (i.e., the ExPEx attribute items) and WCS likeability scores predicted sentencing decision was a good fit and was significant [$\chi_{\left(9\right)}^{2}$ = 36.09, *p* < 0.001], accounting for 20.5% of the variance in sentencing decisions (Nagelkerke *R*^2^ = 0.205). Ratings of the opinion attribute was the only independent predictor of sentencing decision (*p* = 0.045). The remaining predictors were not significant (all *p*s ≥ 0.202 see Table 11).

**Table 11:** Logistic regression predicting sentencing decision from continuous expert persuasion expectancy (ExPEx) ratings and witness credibility score (WCS) for likeability.

	***B***	**SE**	**Wald**	***df***	***p***	**Odds Ratio**	**95% CI for Odds Ratio**	
							**Lower**	**Upper**
Foundation	0.008	0.011	0.52	1	0.473	1.01	0.99	1.03
Field	−0.015	0.012	1.49	1	0.223	0.99	0.96	1.01
Specialty	0.004	0.012	0.08	1	0.775	1.00	0.98	1.03
Ability	−0.002	0.012	0.02	1	0.889	0.998	0.98	1.02
Opinion	0.025^*^	0.013	4.02	1	0.045	1.03	1.00	1.05
Support	−0.012	0.01	1.46	1	0.227	0.99	0.97	1.01
Consistent	0.012	0.012	0.93	1	0.334	1.01	0.99	1.04
Trustworthy	0.013	0.012	1.28	1	0.258	1.01	0.99	1.04
WCS-Likeability	0.026	0.021	1.63	1	0.202	1.03	0.99	1.07

*Life in prison was coded as zero and death was coded as one. B, unstandardised regression coefficient; SE B, standard error of the coefficient; CI, confidence interval*.

* *p < 0.05*,

^**^p < 0.01, ^***^p ≤ 0.001.

### Study 2 Discussion

Study 2 further examined the effect of expert likeability and quality on jurors' perception of expert persuasiveness and sentencing decisions. As in Study 1, we found that participants' perceptions of the persuasiveness of expert evidence were significantly affected by evidence quality and expert likeability. There were also no interactions between evidence quality and likeability. We also found that subjective perceptions of the eight ExPEx attributes and likeability again accounted for approximately 80% of the variance in persuasiveness scores.

While higher quality experts were more persuasive than lower quality experts, Study 2 suggests that adding negative likeability cues reduces perceived likeability and persuasiveness, while adding positive likeability cues did not increase either likeability or persuasiveness. These results replicate the Study 1 finding that the persuasiveness of an expert opinion is determined by both its underlying scientific quality, and superficial aspects of the experts' interpersonal style, but go further to suggest that it may be an unfriendly, arrogant, and conceited style that is particularly influential on persuasiveness.

However, as in Study 1, likeability did not affect sentencing decisions while aspects of evidence quality did. Participants' perceptions of the clarity and conservativeness of the experts' opinion uniquely predicted sentencing outcomes. Likeability condition and ratings did not directly impact sentencing decisions. Thus, concerns about impact of likeability on jurors' sentencing outcomes may be misplaced.

## General Discussion

Two studies examined the effect of expert quality and likeability on potential jurors' perceptions of the persuasiveness expert evidence and sentencing decisions in a Capitol case. Across both our studies we found that higher quality experts were regarded as more persuasive than lower quality experts. We also found that less likeable experts were considered less persuasive than more likeable experts, irrespective of evidence quality. Moreover, models predicting persuasiveness from continuous ratings of expert quality attributes and likeability were significant and accounted for ~80% of the variance in persuasiveness ratings. This result is particularly impressive considering participants were evaluating detailed trial transcripts and videos. Even so, likeability did not significantly affect sentencing outcomes, whereas various elements of expert quality did (i.e., trustworthiness and foundation in Study 1; opinion in Study 2). Models predicting sentencing decisions from continuous ratings of quality and likeability accounted for a smaller but significant 20–30% of the variance.

### Expert Persuasiveness

This research is the first to show that jurors' perceptions of persuasiveness are influenced by expert likeability even in scenarios where very rich information is available about expert evidence quality. This suggests that previously observed likeability effects were not merely an artefact of simplistic or sparse decision-making scenarios. Rather, likeability appears to be genuinely influential in determining how persuasive expert evidence will be.

We also found evidence that the effects of being a dislikeable expert are more impactful than the effects of being likeable. Specifically, we found that a video of an arrogant, conceited, disagreeable expert reduced both likeability and persuasiveness compared to a neutral transcript. But a video of a smiling, modest, open expert did not increase either likeability or persuasiveness compared to a neutral transcript. Thus, irrespective of evidence quality–we saw clear evidence of a dislikeability cost, but we were not able to produce an equivalent likeability benefit. Indeed, in our scenario the cost of being dislikeable was substantial, and in descriptive terms resulted in a low-likeability but *high-quality* expert being treated similarly to a high-likeability but *low-quality* expert.

Our finding that it may be dislikeability rather than likeability that affects persuasiveness is somewhat inconsistent with past research suggesting that likability *boosts* credibility and persuasiveness (Brodsky et al., 2009; Neal et al., 2012). However, this may be because previous studies did not include a neutral likeability control condition as a baseline to gage the effect of likeability manipulations. When this control condition was added, the data clearly suggested the effect of likeability cues was asymmetric and driven by negative rather than positive expert likeability attributes. In fact, our data suggest that likeability ratings may have been at ceiling even in the neutral likeability condition whereby participants seemed to assume the expert was likeable, until proven otherwise. This suggests it may be impractical or at least very difficult for experts to make themselves more likeable than jurors expect but can easily fall short of existing high expectations.

Across both studies we also found strong and consistent evidence that higher quality evidence is more persuasive than lower quality evidence. This result fits with previous research using rich representations of expert opinion quality (Martire et al., 2020) but is somewhat inconsistent with concerns about juror insensitivity to evidence quality (Cooper et al., 1996; Diamond and Rose, 2005; Hans et al., 2007, 2011; McAuliff and Kovera, 2008; McAuliff et al., 2009; Koehler et al., 2016; Eldridge, 2019). In our studies, jurors were provided with information about an expert's field, their specialist background, their proficiency, the validity of their practicing domain, their trustworthiness, consistency with other experts, their supporting evidence and opinion clarity. This level of information is arguably necessary for an informed evaluation of expert quality and exceeds the level of information provided in previous studies that have typically found that jurors struggle to determine evidence quality (Martire et al., 2020). Our results suggest that jurors can appropriately evaluate evidence quality when they have access to more of the relevant information that they need for the task. This interpretation is in line with the ELM perspective of information processing (Petty and Cacioppo, 1984, 1986) whereby decision-makers are more likely to systematically process information if they have sufficient knowledge and capacity (Petty and Cacioppo, 1984, 1986). However, it remains to be seen whether jurors can also use detailed information about expert quality to differentiate between more marginal or subtle differences in evidence quality than those we used in our manipulations. Future research is needed to examine this possibility.

Even so, it is important to note that sensitivity to evidence quality did not remove the effects of dislikeability. When jurors had the information and knowledge to effectively evaluate expert evidence quality, they still used information about expert likeability to determine how much credibility, value, and weight to give the expert evidence. Given that likeability is not related to expert quality or merit, the fact there is a persuasion cost of dislikeability remains problematic, particularly when we see that high-quality evidence is viewed similarly to low-quality evidence from a more likeable expert. Thus, our data show that likeability has the potential to undermine the effects of evidence quality in an undesirable way.

More broadly, across both studies, we found that subjective ratings of evidence quality and likeability impacted persuasiveness. In both studies, we found that subjective ratings of the eight ExPEx attributes and likeability accounted for approximately 80% of the variance in persuasiveness. This suggests that jurors' perceptions of these markers collectively provide a good account of persuasiveness judgments. Impressions of the expert's trustworthiness, their specialist background, their opinion, the consistency of their judgment with other experts, their supporting evidence, and ability were all unique predictors of persuasiveness. This also indicates that jurors use relevant indicators of evidence quality to determine how persuasive an expert will be.

### Sentencing Decisions

Although allocation to expert evidence quality and likeability condition significantly influenced ratings of persuasiveness, this did not translate into a direct impact on sentencing decision. The finding that expert likeability condition did not predict sentencing decision is consistent with the literature examining expert likeability (Brodsky et al., 2009; Neal et al., 2012; Parrott et al., 2015). Therefore, while likeability is considered in judgments of expert persuasiveness, and may make jurors more inclined to agree with the expert, it does not appear to materially affect the final sentencing outcome. Sentencing decisions are consequential and require jurors to consider a wider range of trial considerations relating to the defendant, the sentencing options, and the expert (Greene et al., 2007). Therefore, jurors may pay less attention to likeability in this context, instead focusing on more relevant information (i.e., the expert's opinion, trustworthiness, and foundation).

Although the absence of an effect of quality condition on sentencing can also be attributed to the same broad complexity of sentencing decisions, that explanation seems unsatisfactory in this case. The quality of the expert opinion *should* be a key determinant of the sentencing decision in this trial scenario, even when considering the broader trial context. Specifically, a high-quality expert opinion that is consistent with the application of the death penalty, should result in more death sentences than a low-quality version of the same opinion. The fact that this did not happen even though jurors were more persuaded by high- than low-quality opinions suggests that jurors may not know how to apply low- and high-quality evidence in their sentencing decisions. This might explain why much of the literature suggests that expert evidence is universally persuasive—jurors may be influenced by the expert–but they also may struggle to incorporate evidence quality into their final judgments (Cutler et al., 1989; Ivković and Hans, 2006; Daftary-Kapur et al., 2010; Bornstein and Greene, 2011).

The idea that jurors' may not know how to incorporate evidence quality into their sentencing decisions is further supported by regression models considering continuous ratings of expert quality and likeability. In both studies, we found that subjective ratings of the eight ExPEx attributes and likeability accounted for between 20 and 30% of the variance in sentencing decision. This was substantially less than the variance accounted for by quality and likeability in persuasiveness ratings (~80%). Therefore, perceptions of quality and likeability were less influential for sentencing decisions. This suggests other factors became important or increased in prominence for sentencing that were less relevant to the evaluation of persuasiveness. Indeed, since these new factors appear to be de-emphasising valid quality indicators, it is important to understand what these other factors might be, why they are being used and whether they are logically relevant to sentencing determinations or not. This would form a fruitful line of research to pursue in future studies.

Despite this, we did find that the continuous subjective ratings of foundational validity (Study 1), trustworthiness (Study 1), and opinion (Study 2) were unique predictors of sentencing decision. Likeability ratings were not. This suggests that jurors are incorporating some relevant markers of expert evidence quality into their sentencing decisions. However, these indicators were not consistent across studies, and many valid indicators of quality were not significant independent predictors. Therefore, there is substantial scope for quality information to take a larger role in sentencing decisions and future research should look at methods to improve utilisation of quality information in jurors' sentencing decisions.

### Implications

Altogether, these findings suggest that likeability impacts perceptions of persuasiveness but not by increasing persuasiveness, rather by decreasing it. Experts already appear to be assumed to be likeable at baseline, so attempts to be more likeable may not be effective. Instead, experts should consider whether their highly confident, authoritative, or self-assured interpersonal style could come across as arrogant, disagreeable, or conceited, because being seen in these ways may result in high-quality evidence being discounted to the point where its impact is akin to lower quality evidence provided by a more likeable expert.

More significantly, higher quality evidence was more persuasive than lower quality evidence, irrespective of how likeable an expert was. Sentencing decisions were also affected by perceptions of opinion clarity, discipline foundational validity and trustworthiness. Thus, these results suggest that experts can make their evidence more compelling, and influential by increasing the objective quality of their evidence and communicating that quality to decision-makers.

### Limitations and Future Directions

One limitation of this research is that both studies involved the same Capitol murder case and sentencing decisions. Sentencing is not the only kind of legal decision made by jurors and so it remains to be investigated whether likeability influences jurors' decision-making in other cases and for other types of decisions (e.g., verdicts, liability, damages). Such information is vital to determine the generalisability of our findings. Further, our participants were not assessed for death-qualification (Witherspoon v. Illinois, 391 U.S. 510, 1968). Participants in our study may therefore be more or less willing to impose a death-sentence than real jurors deciding the same case. For this reason, it would be valuable for future research to include questions to establish a death-qualification. However, we note that this is unlikely to affect our results because we were interested in between-group differences in persuasiveness, rather than verdict frequency *per se*.

Another limitation relates to the ecological validity of our study. The mock trial in our first study was a transcript, participation took ~20–40 min including post-trial decision-making, and there was no deliberation phase. This does not reflect real criminal trials which typically are conducted in-person, and can last for weeks or months. Our participants were also predominantly recruited via MTurk and may therefore differ from real jurors in terms of demographic characteristics and investment in the task. To improve the ecological validity, we used videoed trial materials rather than a transcript in Study 2 because it has been suggested that trial videos improve the ecological validity of experimental juror studies (Studebaker et al., 2002). We further ensured higher data quality standards by implementing multiple attention and manipulation check measures, constraining the time allocated to complete the study and narrowing participating criteria to higher-quality respondents. Indeed, per Lieberman et al. (2016), the methodology of the current studies surpasses the acceptable criteria for juror decision-making paradigms. Nonetheless, future studies should consider using longer in-person trials involving more types of evidence and involving jury deliberation.

Finally, it is worth mentioning that participants in our study were asked to complete the evidence quality and likeability measures prior to rating persuasiveness and making sentencing decisions. It is therefore possible that jurors were primed with quality and likeability information that they might not have otherwise considered in their assessments of persuasiveness and sentencing options. We included these measures before the persuasiveness judgment to measure the maximum impact our manipulations might have on perceptions of persuasiveness and sentencing decisions. We reasoned that if there is no effect of quality or likeability under these conditions, then we could not reasonably expect a larger effect of either likeability or quality in real-world settings. That is, we wanted to have the best possible chance of detecting any influence of evidence quality or likeability considerations. The fact that we did not see effects of likeability on sentencing under these conditions strongly suggests that likeability does not significantly affect sentencing decisions. Conversely, the significance of quality attributes suggests that quality may affect real world sentencing decisions. Indeed, the magnitude of the quality effects in our study were consistent with those obtained in other studies where quality was not primed prior to measuring persuasiveness (Martire et al., 2020). Even so, future research could randomise question order to remove any possible priming effects.

## Conclusion

Our results suggest that expert evidence quality and likeability both impact perception of expert persuasiveness. Specifically, dislikeability reduces persuasiveness irrespective of evidence quality. However, only subjective impressions of the foundational validity, trustworthiness, and clarity of the expert opinion significantly predicted Capitol sentencing decisions. Thus, concerns about juror reliance on the peripheral likeability cue may be most relevant to evaluations of the expert evidence in isolation, rather than to trial outcomes. Our results also strongly suggest that likeability does little to boost persuasion while being disliked has a clear cost. Thus, experts can take comfort from the fact that weak evidence is not bolstered by an affable interpersonal style, but they may rightly be concerned that this superficial attribute has the potential to weaken the persuasive power of otherwise high-quality evidence. Thus, care should be taken to ensure that confidence does not become conceit if experts want their evidence to be given its merited value.

## Data Availability Statement

The datasets presented in this study can be found in online repositories. The names of the repository/repositories and accession number(s) can be found at: https://osf.io/yfgke/.

## Ethics Statement

The studies involving human participants were reviewed and approved by University of New South Wales Human Research Ethics Approval Panel C – Behavioural Sciences (#3308). The patients/participants provided their written informed consent to participate in this study.

## Author Contributions

MY and KM contributed to the study concept, experimental design, and reporting. MY led the data collection and analysis. Both authors contributed to the article and approved the submitted version.

## Funding

MY was supported by the Australian Government Research Training Program (RTP).

## Conflict of Interest

The authors declare that the research was conducted in the absence of any commercial or financial relationships that could be construed as a potential conflict of interest.

## Publisher's Note

All claims expressed in this article are solely those of the authors and do not necessarily represent those of their affiliated organizations, or those of the publisher, the editors and the reviewers. Any product that may be evaluated in this article, or claim that may be made by its manufacturer, is not guaranteed or endorsed by the publisher.
